# To culture or not to culture: a snapshot of culture-dependent and culture-independent bacterial diversity from peanut rhizosphere

**DOI:** 10.7717/peerj.12035

**Published:** 2021-09-01

**Authors:** Ankit Hinsu, Ashvin Dumadiya, Anjali Joshi, Rohitkumar Kotadiya, Kavan Andharia, Prakash Koringa, Ramesh Kothari

**Affiliations:** 1 Department of Biosciences, Saurashtra University, Rajkot, India; 2 Department of Animal Biotechnology, Anand Agricultural University, Anand, India

**Keywords:** Microbiome, Metagenome, Culturable diversity, Rhizosphere

## Abstract

**Background:**

Sequencing driven metagenomics studies have been instrumental in various aspects of microbiology including identification of newer taxa. While this culture-independent approach has its own merits and demerits, several studies have focussed on comparing it with traditional culture-dependent (CD) approach. However, most of these comparative studies rely on Sanger sequencing of complete 16S rRNA gene from pure culture colonies to determine the culturable bacterial diversity. This approach undercounts culturable diversity as only fewer isolates are selected, sequenced, and identified.

**Methods:**

In this study, we have used an Illumina based partial 16S sequencing to identify all the microbes growing on the media and directly comparing with its culture-independent (CI) counterpart. Eight different media were used to target different organisms from soil. Diversity on these media were compared with their CI counterpart. The NGS data was analysed using DADA2 to provide more resolution to the data.

**Results:**

In line with studies of similar nature, current study presented higher bacterial diversity in CI approach. However, the current study reflected that a greater number of sequence variants were missed out in CI approach as compared to number of sequence variants shared with CD approach. We observed around 322 (5.98%) ASVs (Amplicon Sequence Variants) exclusively present in CD samples while, 234 (4.35%) ASVs were shared between both approaches. Most of these 322 CD exclusive ASVs were classified as *Enterobacteriaceae* family and *Bacillus* genus, with several ASVs annotated at the species level as well, and these organisms are more commonly observed in soil and were also detected in CI approach. Furthermore, 22 genera were exclusively detected in CD samples, most of which were reported from soil and water.

## Introduction

Microorganisms, particularly bacteria, are the main factors behind variety of metabolic and biogeochemical processes in soil. Microbes interact with plant, get influenced by the plant metabolites and directly or indirectly affect the plant health. Many previous studies have characterized these plant-microbe interactions (Cheng, Zhang & He, 2019). However, the challenging part is to isolate these microbes and characterize them. Majority of the microbes are unculturable under laboratory conditions because of several reasons. Hence, classical microbiological techniques also known as culture-dependent methods are unable to present a complete picture of microbial diversity. On the other hand, advanced methods like metagenomics or culture-independent approach overcome this limitation by directly sequencing the DNA of all the microbes in an environmental sample (Handelsman, 2004). However, many studies have highlighted the fact that even metagenomic approach tends to miss out many of the species (Donachie, Foster & Brown, 2007). Few of these missed out organisms tend to grow well on appropriate media implying that metagenomics misses out some commonly present organisms.

The present study was undertaken to evaluate and compare the bacterial diversity as observed by culture-dependent (CD) and culture-independent (CI) methods from the soil rhizosphere samples of groundnut. The microbes were cultured on eight different media suitable for different types of microbes and were identified by next-generation sequencing (NGS). Unlike the majority of previous studies comparing CD and CI approaches, all the colonies on the plate were sequenced for 16S rRNA using NGS rather than individual colonies using Sanger sequencing.

## Materials & methods

### Sample collection

A total of five samples of groundnut/peanut (*Arachis hypogea*) rhizosphere were collected from two nearby farms each, near Porbandar, Gujarat, INDIA (21.618389 N, 69.866106 E). The samples were collected during the nodulating phase of the crop and named as R3 (Rhizosphere3; from Farm1) and R4 (Rhizosphere4; from Farm2). The samples used in this study are part of another unpublished study on peanut rhizosphere and sample names are used as such. The soil surrounding the plants was carefully removed before uprooting the plants. Plants were then shaken to remove any loosely attached soil. Soil attached on the roots (Rhizosphere) was then collected by washing the roots in 40 ml sterile Normal Saline buffer in sterile 50 ml tubes. Five different plants were uprooted, and samples were collected from them during both sampling. Samples for culture-dependent (CD) approach were transported to lab in cool conditions (at 4–10 °C) and proceeded further next day. Samples for culture-independent (CI) approach were transferred to lab at −20 °C and stored at −80 °C till further processing.

### Sample processing

For CD approach, all five samples from each farm were pooled and diluted serially upto 10^−6^ using sterile water. The last two serial dilutions, 10^−5^ and 10^−6^, were used for spreading and plated on eight different media supplemented with cycloheximide (60 µg/ml of media) as listed in Table 1. The media were selected based on their selective enrichment of common soil organisms as well as their routine use in isolating soil-borne organisms. The plates were incubated at 27 ± 2 °C and 37 ± 2 °C. The spreading of each dilution was done in triplicates for given media and temperature, respectively. After incubation of 14 days, entire plate surface (this should cover all the colonies same or different) was scrapped and collected in Qiagen RNAprotect Bacteria Reagent (Qiagen, Germany). Colonies were pooled in a tube for each media and each farm, *i.e.*, colonies from 12 plates (2 temperatures $\times$ 2 dilutions $\times$ 3 replicates = 12 plates) pooled in a single tube. Total 16 tubes were obtained (2 farms $\times$ 8 media). All the colonies were mixed properly, and DNA was extracted from the pool of colonies using QIAGEN QIAamp DNA Mini Kit (Qiagen, Hilden, Germany) following manufacturer’s protocol of bacterial genomic DNA extraction. Extracted DNA was checked on agarose gel for quality and quantified using Qubit 3.0 (Invitrogen, Waltham, CA, USA).

**Table 1:** List of media used for culture study.

Media	Symbol used for samples	Targeted organisms
R2A agar	R2A	Slow growing bacteria
Soil extract agar	SEA	Common soil bacteria
Actinomycetes isolation agar	AIA	Actinomycetes isolation
Azotobacter mannitol agar	AMA	Azotobacter isolation
Tryptic soy agar	TSA	Fastidious growing organisms
Nutrient agar	NB	General bacteria
YEM agar	YEM	Agrobacterium & soil bacteria
Yeast mannitol agar w/Congo red	YMA	Rhizobium isolation

**Note:**

All the media were procured from HiMedia (Mumbai, India).

For CI approach, all samples were processed separately. Samples were thawed and centrifuged briefly to collect soil at bottom. The buffer was decanted, and remaining soil was mixed to homogenize. DNA was extracted from 0.2 gm homogenized soil using QIAGEN PowerSoil DNA Extraction Kit (Qiagen, Hilden, Germany) following manufacturer’s protocol. Extracted DNA was checked on agarose gel for its quality and quantified using Qubit 3.0 (Invitrogen, Waltham, CA, USA).

### Library preparation and sequencing

DNA from CI approach samples (*n* = 10) and CD approach samples (*n* = 16) were diluted to 5 ng/µl. PCR was performed with diluted DNA using Illumina adapter fused primers 341F and 785R targeting V3–V4 region of 16S rRNA gene as illustrated in Illumina 16S Library Preparation Guide (Illumina, San Diageo, CA, USA) (Klindworth et al., 2013). Further steps were followed as given in the protocol for Illumina 16S Library preparation guide. Prepared libraries were checked on Agilent 2100 Bioanalyser (Agilent, Santa Clara, CA, USA) for their size and quantified on Qubit 3.0 (Invitrogen,Waltham, CA, USA). The CD and CI samples’ libraries were sequenced in separate runs on Illumina MiSeq system using 250 $\times$ 2 v2 paired-end chemistry.

### Data analysis

Generated data was imported in R v3.6.2 environment wherein all the analysis was carried out. Sequences were analyzed using DADA2 package v1.14.0 following analysis pipeline as given in tutorial (https://benjjneb.github.io/dada2/tutorial.html) (Callahan et al., 2016). Since samples of CD and CI were sequenced in separate runs, runs were separately processed till sequence table construction and then merged for further steps as instructed in “big data” tutorial (https://benjjneb.github.io/dada2/bigdata.html). Further, taxonomy was assigned using SILVA v132. The ASV table, assigned taxonomy and sample metadata information were combined as a phyloseq object (phyloseq package version 1.30.0) (McMurdie & Holmes, 2013). This object was then further used for alpha diversity indices estimation, between-sample diversity estimation by Bray–Curtis distance based PCoA, taxonomy agglomeration as well as for presence-absence based comparison. Other R packages used include microbiome v1.8.0 (Lahti & Shetty, 2017), vegan v2.5.6 (Oksanen et al., 2019) and ggpubr v0.2.5 (Kassambara, 2020) with all the plots were made using ggplot2 v3.2.1 (Wickham, 2016).

The complete script/steps for analysis along with the data can be obtained from https://doi.org/10.5281/zenodo.4896308 for reproduction of results. The raw data is also submitted in SRA under the BioProject PRJNA665712. The accession numbers for CD samples are SRR12728805–SRR12728820 and for CI samples are SRR12732125–SRR12732134.

## Results

We generated around 2.2 million paired-end reads for samples from CD-approach (culture-dependent) and 0.7 million reads for samples from CI-approach (culture-independent). DADA2 based pipeline inferred 7,344 ASVs (Amplicon Sequence Variants), out of which 5,381 ASVs having more than five supporting reads were considered for further analyses.

### Alpha diversity

**Figure 1:**
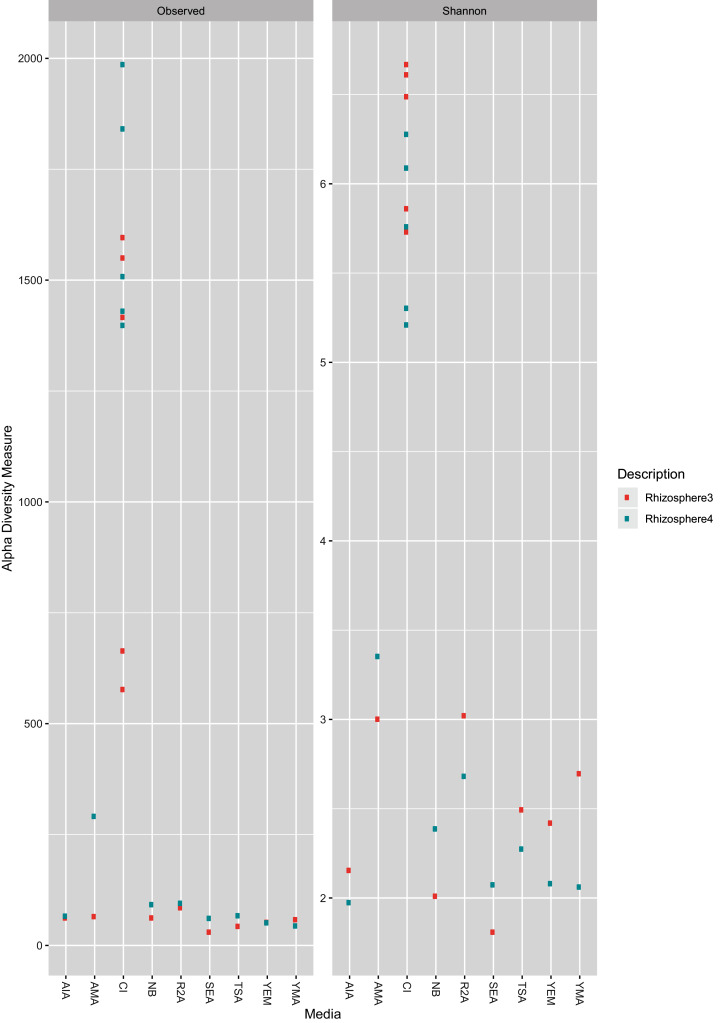
Observed ASV count and Shannon diversity distribution plot. The color is coded for two sample collections used in the study, R3 (Rhizosphere3) and R4 (Rhizosphere4). The X-axis represents either CI approach (marked as CI) or name of media (as given in Table 1).

As expected, the CI approach showed a greater number of ASVs (Wilcoxon test, BH (Benjamini–Hochberg) adjusted *p*-value = 0.00003) as well as higher Shannon Index (Wilcoxon test, BH adjusted *p*-value = 0.0000004) than CD samples (Fig. 1). Further, no significant differences (Wilcoxon test, BH adjusted *p*-value < 0.05) was observed between two farms (R3 and R4; see methods section) for both CD and CI approach. A rarefaction plot also showed asymptotes for all the samples (Fig. S1).

### Taxonomic profile

**Figure 2:**
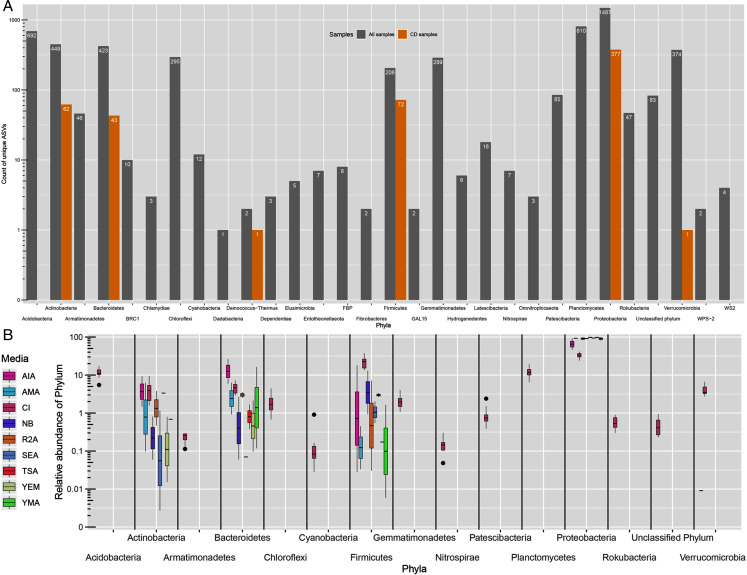
Taxonomic distributions. (A) Phylum-level ASV count distribution in all samples (includes CD and CI samples) and only culture-dependent samples. (B) Relative abundance of top 15 phyla across diﬀerent media. CI represent culture-independent data.

All ASVs were classified as Bacteria. However, 83 ASVs remained unassigned at phylum level. From 29 detected phyla, ASVs from only six phyla namely, Proteobacteria (377), Firmicutes (72), Actinobacteria (62), Bacteroidetes (43), Deinococcus-Thermus (1) and Verrucomicrobia (1) were detected in CD samples (Fig. 2A). Further, the greatest number of ASVs belonged to Proteobacteria phyla (1,487) followed by Planctomycetes (810) and Acidobacteria (692). Proteobacteria was the most dominating phyla in both approaches with 31.5% of average proportion in CI approach while, 46% to 98% in CD approach (Fig. 2B). Majority of the proportion in CD approach samples was of Proteobacteria phylum alone. Other phyla like Acidobacteria, Actinobacteria, Bacteroidetes, Chloroflexi, Firmicutes, Gammatimonadetes, Planctomycetes and Verrucomicrobia were detected with abundance more than 1% in CI approach.

### Presence-absence of ASVs and Genera

**Figure 3:**
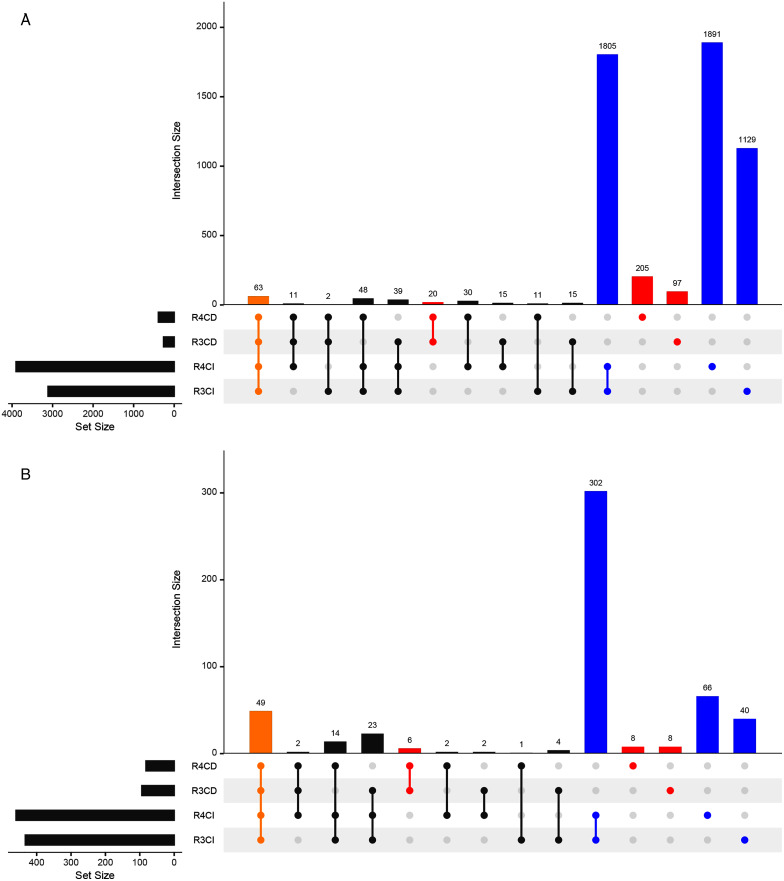
Upset plots representing shared and unique taxas. Upset plot showing distributions of (A) All detected ASVs and (B) Genera-level taxonomy, among culture-dependent and culture-independent sample-groups. Points colored in yellow, red and blue refer to ASVs/genera present in all four groups, CD-exclusive samples and CI-exclusive samples, respectively.

A total of 3,112, 3,902, 262 and 390 ASVs were detected in R3, R4, Cultured-R3 and Cultured-R4 samples, respectively (Fig. 3A, Fig. S2). Out of those ASVs, 1,129, 1,891, 97 and 205 ASVs were exclusively observed in R3, R4, Cultured-R3 and Cultured-R4 samples, respectively. Overall, 322 ASVs (5.98%) were detected exclusively in CD approach compared to 4825 ASVs (89.67%) detected exclusively in CI approach, while only 234 ASVs (4.35%) were shared among both approaches. PERMANOVA test on the Bray–Curtis distance of presence-absence matrix showed that CD and CI group of samples differ significantly (R^2^ = 0.37505, *P* = 0.001) while lesser significant differences were observed between collections (R^2^ = 0.08435, *P* = 0.049). The same result when plotted through PCoA also highlighted these differences (Fig. S3). Taxonomically, CD-exclusive ASVs belonged to Proteobacteria (226), Firmicutes (42), Actinobacteria (38), Bacteroidetes (15) and Verrucomicrobia (1) (Fig. S4). At genus level, *Bacillus* (9%) was the most dominant followed by *Pseudomonas* (3%), *Enterobacter* (2%), *Nocardioides* (2%), *Streptomyces* (2%), *Paenibacillus* (2%), *Allorhizobium*-*Neorhizobium*-*Pararhizobium*-*Rhizobium* (2%). Further, many of these ASVs were also assigned at the species level as well, for example, *Bacillus endophyticus*, *Bacillus subtilis*, *Bacillus infantis*, *Pseudomonas indica*, *Pseudomonas stutzeri*, *Pseudomonas taiwanensis* and many others. All these genera were also observed in notable amount in CI approach. However, many of the species were not observed. Therefore, to provide more leniency in thresholds, we also compared samples at genus level to find if any genera are exclusively detected in CD samples only.

Around 433, 460, 94 and 82 genera were detected in R3, R4, Cultured-R3 and Cultured-R4 samples, respectively from 527 unique genera (Fig. 3B). Forty-nine genera were common among all four groups while, 22 and 408 genera were exclusively present in CD and CI groups, respectively. These 22 genera were *Aeromonas*, *Agrococcus*, *Amycolatopsis*, *Arthrobacter*, *Cellulomonas*, *Comamonas*, *Cronobacter*, *Curtobacterium*, *Georgenia*, *Kocuria*, *Luteibacter*, *Methylobacterium*, *Methylophilus*, *Micrococcus*, *Nocardiopsis*, *Prauserella*, *Prevotella*_1, *Promicromonospora*, *Rheinheimera* and unknown genera from families *Intrasporangiaceae*, *p-251-o5* and *Planococcaceae*. A total of Nine of these genera were detected on more than one culture media. Also, majority of these genera belonged to Actinobacteria phylum (12), followed by Proteobacteria (7: 6 Gammaproteobacteria and 1 Alphaproteobacteria), Bacteroidetes (2) and Firmicutes (1).

## Discussion

The work by Razumov in 1932 is perhaps the first report showing that limited proportion of microbial world is cultivable. This experiment was later on termed as “the great plate count anomaly” by Staley and Konopka in 1985 (Carini, 2019; Reguera, 2016). It was realized that not all microbes can be cultured under the laboratory conditions probably because of their specific growth requirements. This led to development of several molecular methods for the study of microorganisms including Random Amplified Polymorphic DNA (RAPD), PCR-RFLP (Restriction Fragment Length Polymorphism), DNA Fingerprinting, Stable Isotope Probing, Terminal-Restriction Fragment Length Polymorphism, Fluorescence *in Situ* Hybridization, and Microarray (Zhao et al., 2011).

Apart from these, another method called ‘metagenomics’ was developed wherein entire nucleic acid from the community was studied most commonly through next-generation sequencing (Handelsman, 2004). Originally, metagenomics did start from study of soil microbes and currently it is applied in various fields of research in the biological sciences including human health (Garrido-Cardenas & Manzano-Agugliaro, 2017). Although, metagenomics is extremely popular, it also fails to reflect the true diversity present in the sample due to some of its limitations. For example, bias in the sample collection, processing, various DNA extraction methods/protocol, PCR primers and conditions used to amplify the target, data analysis pipeline and database all these affect the final interpretation of the results (Laudadio et al., 2019). Apart from this, several studies/researchers including the pioneering study by the Kaiser, Puhler & Selbitschka (2001) have reported the limitation of metagenomics in missing out several organisms including the culturable species (Donachie, Foster & Brown, 2007; Kaiser, Puhler & Selbitschka, 2001; Lee et al., 2016; Quere et al., 2019; Tytgat et al., 2014; Yashiro, Spear & McManus, 2011). However, in all these studies, in the culture dependent part, bacteria were isolated in their pure form and then identified them through Sanger sequencing. Another study by Lagier et al. (2012) demonstrated the first-ever “culturomics” based approach for the large scale cultivation of microbes by tweaking the media compositions and culture conditions while using GC-MS (Gas chromatography–mass spectrometry) as identification for differentiating colonies.

Most of these studies reported that CI approach had higher diversity than CD approach. Similar conclusions can also be drawn from present study as well. However, most of these studies have considered only selected differentiating isolates from CD approach which provides an incomplete picture of culture-dependent diversity. We tried to circumvent this problem by scrapping-off all the colonies from the media and opting for NGS-based metagenomics like identification. This should reflect almost entire cultivable-diversity including several microcolonies. Further, we sequenced portion of 16S rRNA gene similar to metagenomics rather than complete 16S as performed in all other studies. By doing so, we believe to have included sequences from all the organisms cultivated rather than introducing selection biases based on observation. Further, using diverse set of media, generating high amount of data, and considering enough reads for the analysis, can help provide the complete picture. As per our knowledge, similar approach has also been successfully applied by Zehavi, Probst & Mizrahi (2018) for the study of ruminal microbiota. This approach could be useful when conducting presence-absence based study and not suitable when trying to analyze the abundance of cultivable organisms. Additionally, it would not be possible to isolate and purify the colony on the media if needed for further experiments.

In this study, we could identify 322 ASVs exclusively present in CD samples. Surprisingly, these ASVs were identified in the CI approach as well. However, not all the species level assignments could be observed in CI approach. This could possibly be due to presence of these organisms in very low abundance. Also, since the rarefaction plot shows asymptote for both groups of samples, it can be said that under-sampling or sub-sampling or insufficient reads could not be the reason for obtaining CD-exclusive ASVs. On other hand, comparing only the genera also revealed 22 genera exclusively present in CD samples. Most of these genera are commonly found in soil, forest and water sources including marine (Aamot, Hofgaard & Lysoe, 2017; Bennur et al., 2015; Chase et al., 2016; Hayashi et al., 2018; Kumari, Singh & Lal, 2016; Li et al., 2020; Orsini et al., 2014; White et al., 2018; Zheng et al., 2017). However, few of the genera like *Kocuria*, *Cronobacter*, *Aeromonas* are also linked to human infection as per some recent studies (Kandi et al., 2016; Parra-Flores et al., 2018). Also, surprising was the detection of strict anaerobes like *Prevotella*_1 which are most abundant in human and animal gut (Pandit et al., 2018); and presence of genera from *p-251-o5* family which is a candidatus taxa, taxa with no cultured representative as of yet, reported from animal gut.

The reason for such observations can be associated with multiple reasons. The most common and accepted reason being very lesser abundance of these missed-out organisms in environment such that they are missed out during metagenomic approach. Additionally, the reason could also be attributed to the random chance, sampling error and/or complications arising during DNA extraction for such lesser abundant organisms. Use of different DNA extraction kits and methods depending on the sample source can also introduce little biases in the comparisons. However, rarefaction curves-based support of sufficient sequencing depth coupled with capabilities of DADA2 analysis pipeline in resolving organisms at species/strain level by looking at single nucleotide changes can be the reason why ASV belonging to the same organism can be present/absent in CI approach.

## Conclusions

In all, the current study demonstrated the limitations of CI approach to cover all the organism similar to previous studies. The main purpose of the study however was to reflect on proportion of the actual sequence variants missed out, which is more than what was commonly observed in both the approach. However, the CD-based approach described in this study is limited by the number of medias that can be used for isolation as well as inability to identify and obtain colony of any organisms. Nonetheless, this approach reveals a more accurate comparison of CD and CI approaches.

## Supplemental Information

10.7717/peerj.12035/supp-1 Supplemental Information 1 Rarefaction plot. Rarefaction plot of Observed ASVs (Y-axis) against number of reads (X-axis). The samples are colored by media or as CI (Culture-independent).

10.7717/peerj.12035/supp-2 Supplemental Information 2 Heatmap showing presence-absence of ASVs. Colour represents presence while no colour (white colour) represents absence of ASVs. Top section of ASVs are observed exclusively in culture-dependent data.

10.7717/peerj.12035/supp-3 Supplemental Information 3 PCoA plot of samples. PCoA plot based on Bray-Curtis distance on presence-absence matrix of ASVs.

10.7717/peerj.12035/supp-4 Supplemental Information 4 Krona plot representing CD-exclusive diversity. Snapshot of Krona plot representing diversity of ASVs exclusively detected in culture-dependent data.

## Additional Information and Declarations

### DNA Deposition

The following information was supplied regarding the deposition of DNA sequences: The raw data are available in SRA under BioProject PRJNA665712; CD samples: SRR12728805– SRR12728820; CI samples: SRR12732125–SRR12732134.

### Data Availability

The following information was supplied regarding data availability: The complete script/steps for analysis along with the data are available at Zenodo: ankit4035. (2021). ankit4035/CD-CI: (1.0.1). Zenodo. DOI 10.5281/zenodo.4896308.

## References

[ref-1] Aamot HU, Hofgaard IS, Lysoe E (2017). Complete genome sequence of Luteibacter rhizovicinus strain LJ96T, isolated from the rhizosphere of barley (Hordeum vulgare L.) in Denmark. Genom Data.

[ref-2] Bennur T, Kumar AR, Zinjarde S, Javdekar V (2015). Nocardiopsis species: incidence, ecological roles and adaptations. Microbiological Research.

[ref-3] Callahan BJ, McMurdie PJ, Rosen MJ, Han AW, Johnson AJ, Holmes SP (2016). DADA2: high-resolution sample inference from Illumina amplicon data. Nature Methods.

[ref-4] Carini P (2019). A cultural renaissance: genomics breathes new life into an old craft. mSystems.

[ref-5] Chase AB, Arevalo P, Polz MF, Berlemont R, Martiny JB (2016). Evidence for ecological flexibility in the cosmopolitan genus curtobacterium. Frontiers in Microbiology.

[ref-6] Cheng YT, Zhang L, He SY (2019). Plant-microbe interactions facing environmental challenge. Cell Host & Microbe.

[ref-7] Donachie SP, Foster JS, Brown MV (2007). Culture clash: challenging the dogma of microbial diversity. ISME Journal.

[ref-8] Garrido-Cardenas JA, Manzano-Agugliaro F (2017). The metagenomics worldwide research. Current Genetics.

[ref-9] Handelsman J (2004). Metagenomics: application of genomics to uncultured microorganisms. Microbiology and Molecular Biology Reviews.

[ref-10] Hayashi K, Busse HJ, Golke J, Anderson J, Wan X, Hou S, Chain PSG, Prescott RD, Donachie SP (2018). Rheinheimera salexigens sp. nov., isolated from a fishing hook, and emended description of the genus Rheinheimera. International Journal of Systematic and Evolutionary Microbiology.

[ref-11] Kaiser O, Puhler A, Selbitschka W (2001). Phylogenetic analysis of microbial diversity in the rhizoplane of oilseed rape (Brassica napus cv. Westar) employing cultivation-dependent and cultivation-independent approaches. Microbial Ecology.

[ref-12] Kandi V, Palange P, Vaish R, Bhatti AB, Kale V, Kandi MR, Bhoomagiri MR (2016). Emerging bacterial infection: identification and clinical significance of Kocuria species. Cureus.

[ref-13] Kassambara A (2020). Ggpubr: ‘ggplot2’ based publication ready plots. https://CRAN.R-project.org/package=ggpubr.

[ref-14] Klindworth A, Pruesse E, Schweer T, Peplies J, Quast C, Horn M, Glockner FO (2013). Evaluation of general 16S ribosomal RNA gene PCR primers for classical and next-generation sequencing-based diversity studies. Nucleic Acids Research.

[ref-15] Kumari R, Singh P, Lal R (2016). Genetics and genomics of the genus amycolatopsis. Indian journal of microbiology.

[ref-16] Lagier JC, Armougom F, Million M, Hugon P, Pagnier I, Robert C, Bittar F, Fournous G, Gimenez G, Maraninchi M, Trape JF, Koonin EV, La Scola B, Raoult D (2012). Microbial culturomics: paradigm shift in the human gut microbiome study. Clinical Microbiology and Infection.

[ref-17] Lahti L, Shetty S (2017). Tools for microbiome analysis in R Version 2.1.26. http://microbiome.github.com/microbiome.

[ref-18] Laudadio I, Fulci V, Stronati L, Carissimi C (2019). Next-generation metagenomics: methodological challenges and opportunities. Omics-a Journal of Integrative Biology.

[ref-19] Lee SA, Park J, Chu B, Kim JM, Joa JH, Sang MK, Song J, Weon HY (2016). Comparative analysis of bacterial diversity in the rhizosphere of tomato by culture-dependent and-independent approaches. Journal of Microbiology.

[ref-20] Li Q, Ruckert C, Li G, Huang P, Schneider O, Kalinowski J, Jiang Y, Zotchev SB, Jiang C (2020). Prauserella flavalba sp. nov., a novel species of the genus Prauserella, isolated from alkaline soil. International Journal of Systematic and Evolutionary Microbiology.

[ref-21] McMurdie PJ, Holmes S (2013). phyloseq: an R package for reproducible interactive analysis and graphics of microbiome census data. PLOS ONE.

[ref-22] Oksanen J, Blanchet FG, Friendly M, Kindt R, Legendre P, McGlinn D, Minchin P, O’Hara RB, Simpson GL, Solymos P, Stevens MHH, Szoecs E, Wagner H (2019). Vegan: community ecology package. https://CRAN.R-project.org/package=vegan.

[ref-23] Orsini J, Tam E, Hauser N, Rajayer S (2014). Polymicrobial bacteremia involving comamonas testosteroni. Case Reports in Medicine.

[ref-24] Pandit RJ, Hinsu AT, Patel SH, Jakhesara SJ, Koringa PG, Bruno F, Psifidi A, Shah SV, Joshi CG (2018). Microbiota composition, gene pool and its expression in Gir cattle (Bos indicus) rumen under different forage diets using metagenomic and metatranscriptomic approaches. Systematic and Applied Microbiology.

[ref-25] Parra-Flores J, Cerda-Leal F, Contreras A, Valenzuela-Riffo N, Rodriguez A, Aguirre J (2018). Cronobacter sakazakii and microbiological parameters in dairy formulas associated with a food alert in Chile. Frontiers in Microbiology.

[ref-26] Quere G, Intertaglia L, Payri C, Galand PE (2019). Disease specific bacterial communities in a Coralline algae of the Northwestern Mediterranean Sea: a combined culture dependent and-independent approach. Frontiers in Microbiology.

[ref-27] Reguera G (2016). “The great plate count anomaly” that is no more. Company of Microbes.

[ref-28] Tytgat B, Verleyen E, Obbels D, Peeters K, De Wever A, D’Hondt S, De Meyer T, Van Criekinge W, Vyverman W, Willems A (2014). Bacterial diversity assessment in Antarctic terrestrial and aquatic microbial mats: a comparison between bidirectional pyrosequencing and cultivation. PLOS ONE.

[ref-29] White RA, Gavelis G, Soles SA, Gosselin E, Slater GF, Lim DSS, Leander B, Suttle CA (2018). The complete genome and physiological analysis of the microbialite-dwelling Agrococcus pavilionensis sp. nov; reveals genetic promiscuity and predicted adaptations to environmental stress. Frontiers in Microbiology.

[ref-30] Wickham H (2016). Ggplot2: elegant graphics for data analysis.

[ref-31] Yashiro E, Spear RN, McManus PS (2011). Culture-dependent and culture-independent assessment of bacteria in the apple phyllosphere. Journal of Applied Microbiology.

[ref-32] Zehavi T, Probst M, Mizrahi I (2018). Insights into culturomics of the Rumen microbiome. Frontiers in Microbiology.

[ref-33] Zhao L, Ma Z, Luan Y, Lu A, Wang J, Pan L (2011). Molecular methods of studying microbial diversity in soil environments.

[ref-34] Zheng W, Li D, Zhao J, Liu C, Zhao Y, Xiang W, Wang X (2017). Promicromonospora soli sp. nov., a novel actinomycete isolated from soil. International Journal of Systematic and Evolutionary Microbiology.

