# Supplementary figures and images for: To culture or not to culture: a snapshot of culture-dependent and culture-independent bacterial diversity from peanut rhizosphere
Source: PeerJ. 2021 Sep 1;9:e12035. doi: 10.7717/peerj.12035 (PMC8418214; doi:10.7717/peerj.12035)

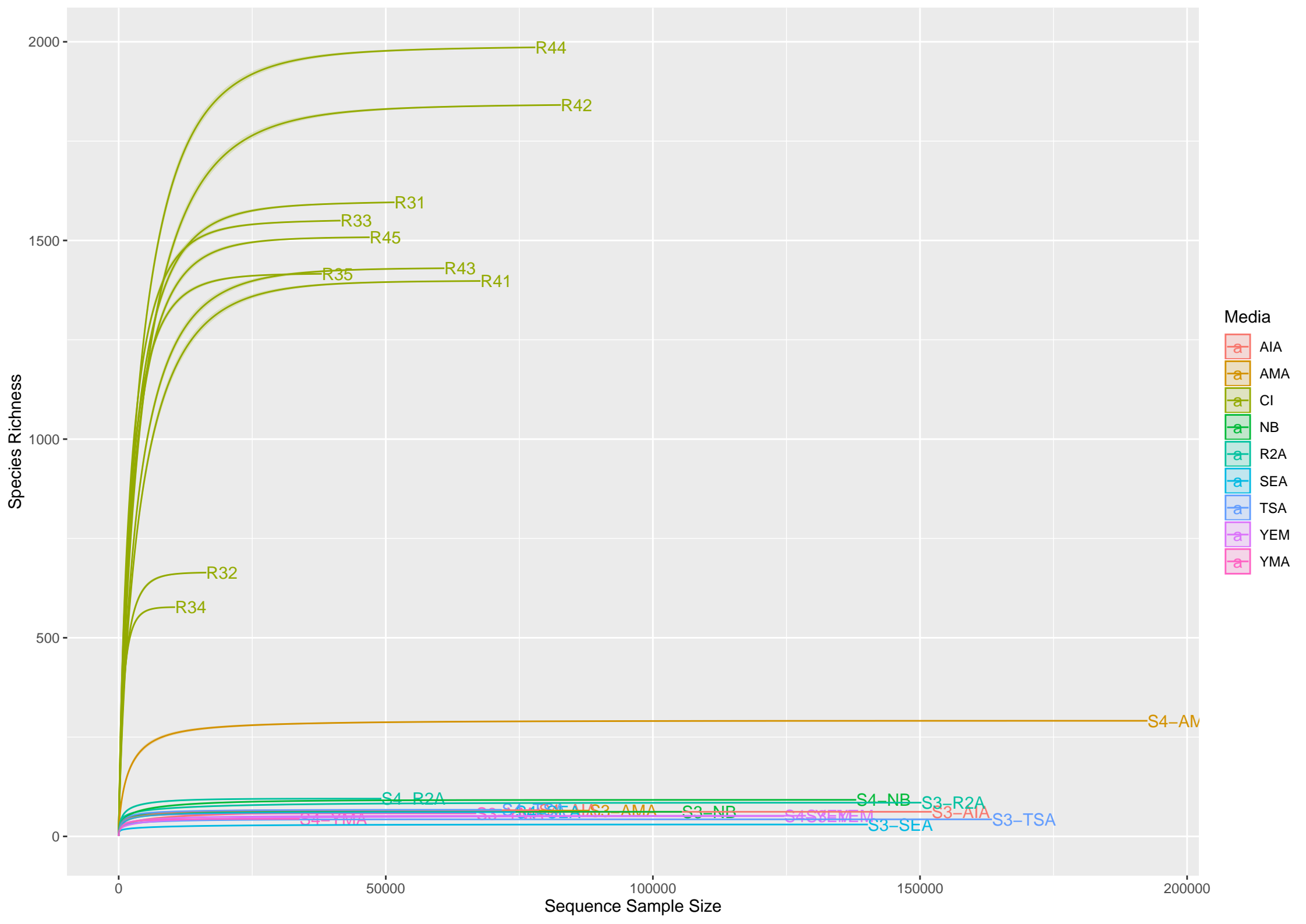

Supplement: Supplemental Information 1 — Rarefaction plot of Observed ASVs (Y-axis) against number of reads (X-axis). The samples are colored by media or as CI (Culture-independent). [file peerj-09-12035-s001.pdf]

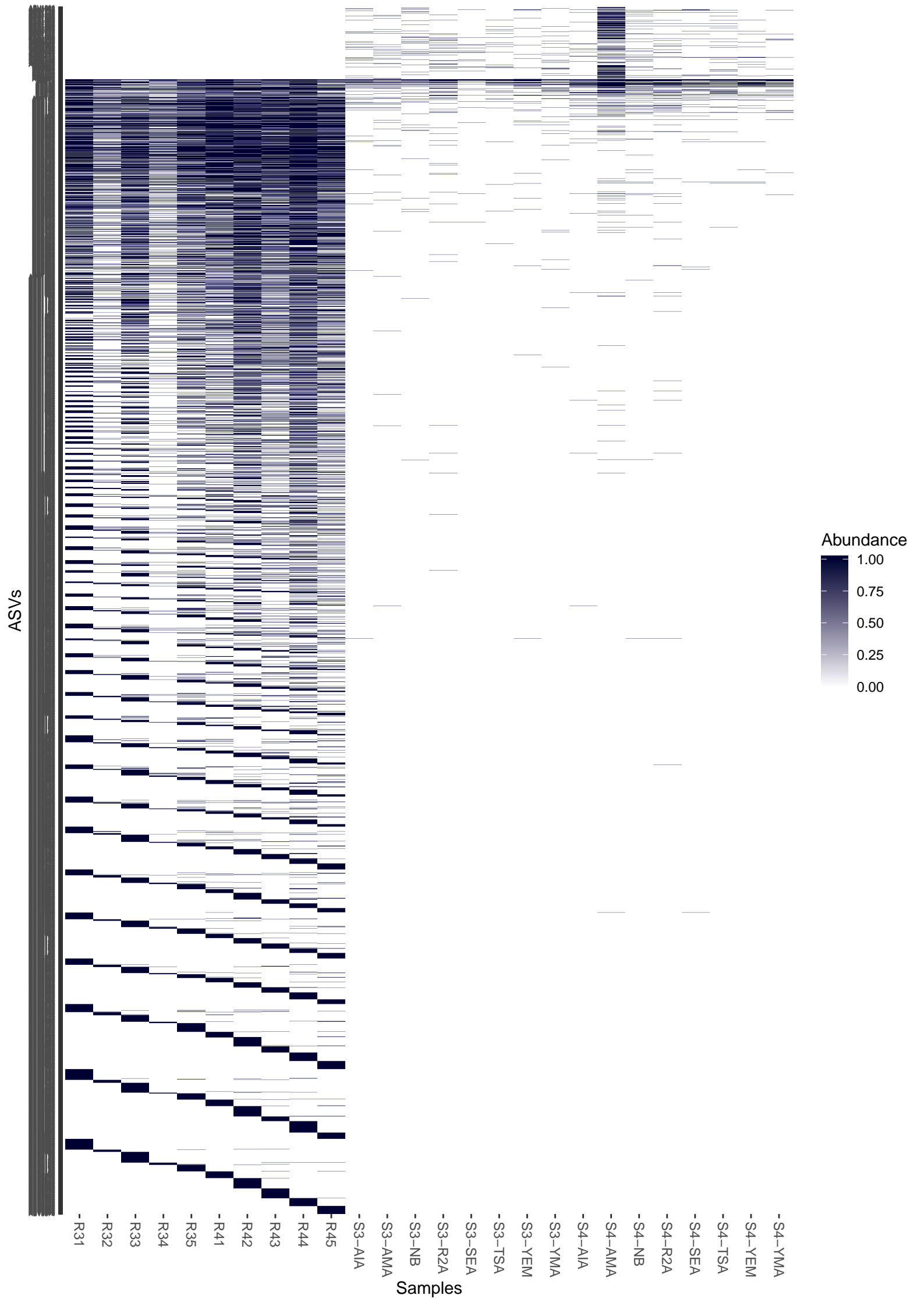

Supplement: Supplemental Information 2 — Colour represents presence while no colour (white colour) represents absence of ASVs. Top section of ASVs are observed exclusively in culture-dependent data. [file peerj-09-12035-s002.pdf]

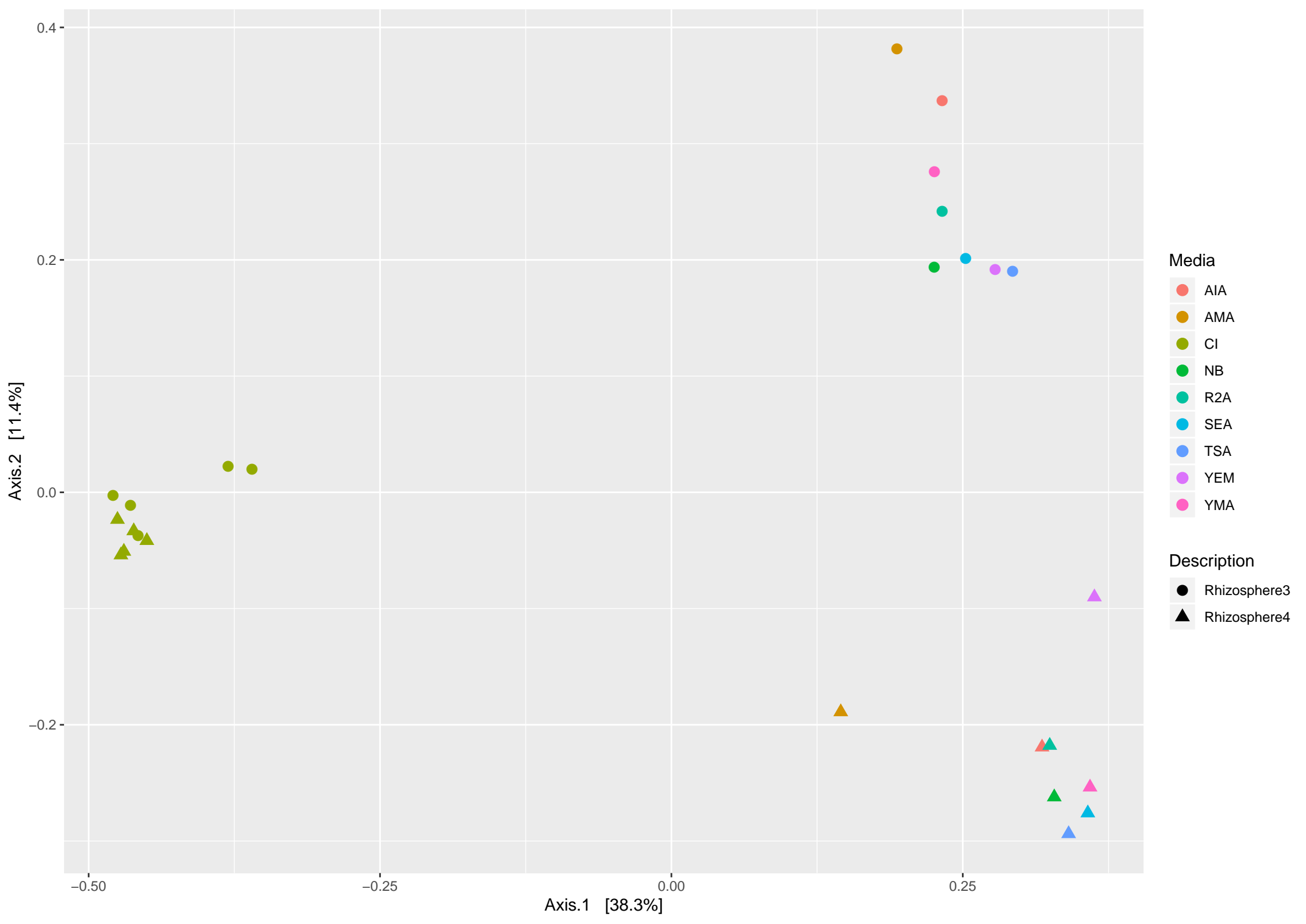

Supplement: Supplemental Information 3 — PCoA plot based on Bray-Curtis distance on presence-absence matrix of ASVs. [file peerj-09-12035-s003.pdf]

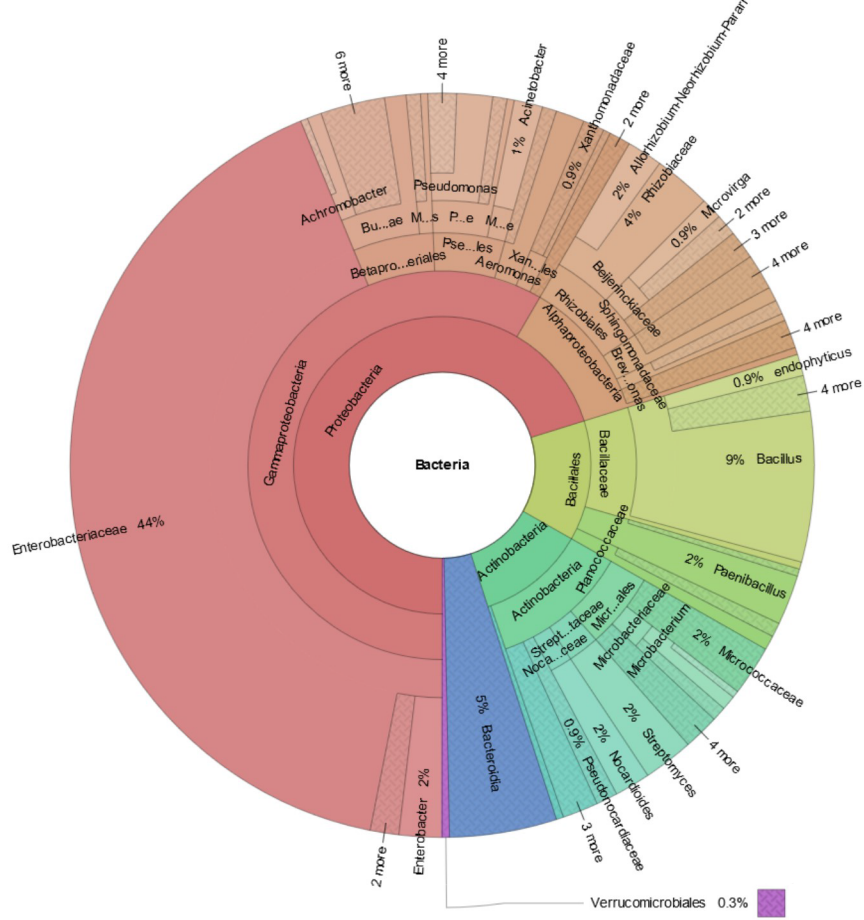

Supplement: Supplemental Information 4 — Snapshot of Krona plot representing diversity of ASVs exclusively detected in culture-dependent data. [file peerj-09-12035-s004.pdf]
